# Co-operation and conflict under hard and soft contracting regimes: case studies from England and Wales

**DOI:** 10.1186/1472-6963-13-S1-S7

**Published:** 2013-05-24

**Authors:** David Hughes, Pauline Allen, Shane Doheny, Christina Petsoulas, Peter Vincent-Jones

**Affiliations:** 1 Department of Public Health & Policy Studies, Swansea University, UK; 2 Department of Health Services Research and Policy, LSHTM, UK; 3 School of Planning and Geography, Cardiff University, UK; 4 School of Law, University of Sheffield, UK

## Abstract

**Background:**

This paper examines NHS secondary care contracting in England and Wales in a period which saw increasing policy divergence between the two systems. At face value, England was making greater use of market levers and utilising harder-edged service contracts incorporating financial penalties and incentives, while Wales was retreating from the 1990s internal market and emphasising cooperation and flexibility in the contracting process. But there were also cross-border spill-overs involving common contracting technologies and management cultures that meant that differences in on-the-ground contracting practices might be smaller than headline policy differences suggested.

**Methods:**

The nature of real-world contracting behaviour was investigated by undertaking two qualitative case studies in England and two in Wales, each based on a local purchaser/provider network. The case studies involved ethnographic observations and interviews with staff in primary care trusts (PCTs) or local health boards (LHBs), NHS or Foundation trusts, and the overseeing Strategic Health Authority or NHS Wales regional office, as well as scrutiny of relevant documents.

**Results:**

Wider policy differences between the two NHS systems were reflected in differing contracting frameworks, involving regional commissioning in Wales and commissioning by either a PCT, or co-operating pair of PCTs in our English case studies, and also in different oversight arrangements by higher tiers of the service. However, long-term relationships and trust between purchasers and providers had an important role in both systems when the financial viability of organisations was at risk. In England, the study found examples where both PCTs and trusts relaxed contractual requirements to assist partners faced with deficits. In Wales, news of plans to end the purchaser/provider split meant a return to less precisely-specified block contracts and a renewed concern to build cooperation between LHB and trust staff.

**Conclusions:**

The interdependency of local purchasers and providers fostered long-term relationships and co-operation that shaped contracting behaviour, just as much as the design of contracts and the presence or absence of contractual penalties and incentives. Although conflict and tensions between contracting partners sometimes surfaced in both the English and Welsh case studies, cooperative behaviour became crucial in times of trouble.

## Introduction

This paper examines NHS contracting in England and Wales in 2008-10, at a time when widening policy differences between the two countries created a natural experiment involving what at face value were respectively ‘hard’ and ‘soft’ versions of the internal market. We put forward three related propositions about contracting in public-sector quasi-markets that are consistent with the findings of our empirical study. We contend that policy designs that emphasize hard market levers, such as fully-specified (‘complete’) contracts, formal dispute resolution procedures, financial penalties and pay-for-performance incentives, underestimate the importance of informal social norms and long-term relationships in real markets, and risk manufacturing an artificial market environment in which actors fall back on informal relationships to keep the system working. Because ongoing relationships and a flexible interpretation of rules are crucial under both hard and soft contracting regimes, the extent of on-the-ground operational differences between England and Wales was smaller than headline policy statements might suggest. Moving forward, the tendency of policy makers to neglect co-operation and organisational norms, and to push instead for a strengthening of competition and market incentives, as has occurred in England, threatens to dismantle the relational networks that keep the NHS working and may de-stabilise the system.

When the Labour Party returned to power at the 1997 general election there was an initial move away from the language of markets and competition that had characterised the early years of the NHS internal market, and less emphasis than before on contracts as instruments for managing provider performance. However, devolution and the creation of national assemblies in Wales, Scotland and Northern Ireland opened a space for policy divergence, and by about 2002 English policy makers, concerned that increased public spending had not yielded commensurate service improvements, again turned to market ideas to reinvigorate the NHS in England. The ‘supply-side market’ that emerged was constructed from five interlocking components: foundation trust hospitals with increased operational autonomy; arm’s-length regulatory bodies independent from the Department of Health (DH) to oversee providers; greater use of private-sector providers for elective surgery; a new ‘payment-by-results’ (PbR) financing system that allowed any provider willing to undertake NHS work at national tariff prices to compete to treat NHS patients, and the right of patients needing planned surgery to choose the hospital that would perform and be paid for this treatment. But while England moved swiftly to implement these reforms, the devolved governments in the other home countries found this path unattractive. Northern Ireland stayed close to the *status quo ante*, while Scotland opted to end the purchaser/provider split and establish unified health boards. In the period covered by this research Wales continued with a soft version of the internal market, and since then has followed Scotland’s lead by abolishing the purchaser/provider split and merging the old local health boards (LHBs) and NHS trusts into unified boards.

Yet despite headline policy differences, there were striking similarities of approach in many areas of contracting practice in England and Wales. Many elements of the technology of contracting and the culture of management spilled over from one system to the other. Both countries utilised national template contracts which imposed a measure of standardisation and set limits on variation in local agreements. While England tended to use health resource groups and provider spells as contract currencies, against Wales’ average specialty prices and deaths and discharges, we found that English organisations did not always reimburse strictly on PbR tariff and that price negotiation and block contracts could still appear in the English system, as they did in Wales. The two systems utilised broadly similar risk management and demand management strategies, and similar dispute resolution arrangements applied.

Relational contract theory [1] predicts that it is behaviour rather than rules that shapes contractual relations. This helps explain the many similarities in contracting practices in England and Wales, and also the occasional deviation from official policy.

## Methods

This study was undertaken during a period of considerable turbulence in the NHS, which changed the nature of purchaser/provider relationships in both countries, and forced changes in the original research aims. What had been conceived as a straightforward comparison of contracting practice in contrasting national cases, ended up providing a snapshot of two systems in transition and raised the question of what contracting practices in the late 2000s might teach us about the prospects of the latest NHS reforms.

The study centred on two English and two Welsh case studies, which each examined relations between a local purchaser and the associated secondary care providers (the NHS hospital trusts in that locality). In one English case study (Beta), we included two primary care trusts (PCTs) that had coordinated their purchasing and worked with the same cluster of providers. The case study sites selected included one predominantly urban and one mixed urban/rural area in each country. Additional data were gathered on agencies from the wider regulatory environment, such as the Care Quality Commission, Monitor, Health Commission Wales and Healthcare Inspectorate Wales.

The research took a similar approach to other recent ‘policy ethnographies’ that utilise a combination of non-participant observation, interviews and documentary analysis to examine NHS management processes [2-4]. We observed a variety of meetings involving *inter alia* contract negotiations and monitoring, individual patient commissioning, waiting list management and NHS reconfiguration (see: Table 1). Interviews were completed with purchaser and provider staff involved in contracting, including chief executives and finance directors, and also with staff in Strategic Health Authorities (SHAs) and Welsh regional offices (see: Table 2). Most meetings and all interviews were tape recorded and fully transcribed. In addition, we scrutinised relevant policy documents, including English and Welsh standard contract templates, individual PCT/trust and LHB/trust contracts, annual operating frameworks, and various national guidelines, commissioning intentions, financial statements and local targets.

**Table 1:** Meetings observed

	England		Wales	
	Case Study Alpa	Case Study Beta	Case Study Delta	Case Study Gamma

Contracting Meetings	22	10	7	5
Quality	3	1		
Individual Patient Commissioning			11	
Cross-border Commissioning			1	1
Reconfiguration/ finance/demand management/clinical networks				16

Note. In Wales, regular contracting meetings were suspended with the impending restructuring of the service, and instead we observed meetings concerned with planning the unified health board framework that would replace contracting. In the text the organisations within Alpha case study, are labelled as Alpha PCT, Alpha trust1, Alpha trust2 and so on, to indicate that their association.

**Table 2:** Interviews completed

	England		Wales	
	Case Study Alpha	Case Study Beta	Case Study Delta	Case Study Gamma

Purchaser managers	4	4	13	9
Trust managers	6	8	8	6
SHA, regional office, regional commissioning support unit	1	1	3	4

Note: Because the Welsh NHS was undergoing fundamental restructuring we also completed a further 21interviews with politicians, special advisors, senior civil servants, officials in Health Commission Wales and Healthcare Inspectorate Wales, and doctors managing the clinical networks.

The study aimed to cover two contracting cycles, spread over the 2008-09 and 2009-10 financial years. This was achieved with English case study Alpha and the two Welsh case studies (Delta and Gamma). Observations for English case study Beta extended only over the 2009/10 year, but the interviews gathered data on the two year period.

Overall then, the fieldwork in England was more narrowly focused on the routine work of contracting, while that in Wales was concerned with reporting how commissioning was being taken forward in a system where routine contracting work was winding down and organisations were about to be restructured. Interviews in both countries were carried out to supplement the observations, and also to provide information on the wider service environments.

## The social embedding of economic activity

The analysis developed here follows a well-established strand of social theory that insists that economic activity is not shaped by individual utility-maximising behaviour alone, but also by social norms and relationships. This is common theme that binds together writings as diverse as Durkheim’s analysis of the normative basis of contracts [5], Polanyi’s ‘double movement’ whereby swings towards unrestrained markets spark corrective attempts to re-build social cohesion, and Granovetter’s work on the ‘smoothing role of social relations in the market’ [7][p.501]. The idea that social relations and norms that discourage malfeasance can bring order to economic life provides a powerful counterpoint to the argument of senior policy advisors in England that incentives should be re-designed so as to harness and control self-interested behaviour [8,9]. Granovetter’s analysis suggests that where problems arise it may be interpersonal relationships rather than economic incentives that allow organisations to keep working smoothly, and that reforms that undermine existing relational networks may have highly negative consequences [7].

The socio-legal theorist Ian Macneil argues in a similar vein that exchange transactions necessarily occur within a ‘social matrix’ and follow characteristic ‘relational patterns’[1][p.344-5]. Macneil suggests that contracts between modern enterprises are almost invariably part of a longer-term relationship between the parties, so that an economic analysis based on rational calculations of advantage in single, ‘discrete’ exchanges has limited utility in explaining real-world behaviour. The formal agreements incorporated in contracts do provide some safeguards for the parties and all contracts rest upon norms of planning, precision and completeness (‘presentiation’) associated with the classical conception of the contract as a statement of the conditions governing the transaction. But Macneil contends that there are also important ‘relational’ norms such as flexibility, solidarity and reciprocity, which derive from the economic and social context of the transaction [1,10]. Here his position comes close to Williamson’s contention that where long-term contracts are involved, and in a context characterised by high specificity of assets, successful relationships depend on a high degree of co-operation and trust [11,12]. Macneil predicts that both discrete and relational norms are involved to a greater or lesser degree, and that though there are likely to be shifts in the balance over time, an excessive swing in one direction will cause problems. Thus a greater concentration on the discrete norms of contract planning and ‘completeness’ – by attempting to draft a ‘hard’ contract that builds in appropriate incentives, fixes responsibilities, specifies what happens when unexpected events arise, and is rigorously enforced – might be predicted to lead to conflictual or adversarial relations with high costs to both parties.

It must be acknowledged that, though they share much common ground, scholars who have examined the arguments about social embedding disagree on many important issues [13]. Over time there are likely to be junctures when both market levers and co-operative behaviour are brought into play. In terms of Macneil’s contractual norms, there are moments when discrete norms concerned with completeness and formal enforcement come to the fore, while at other points co-operation and trust are important. Because Macneil accepts that actors may put more or less emphasis on discrete versus relational norms at different times his theory is not easily falsified, and what this paper offers is less a test of the theory than an exemplification of how far it fits with the empirical data collected. The sections that follow document a number of observed instances where NHS purchasers and providers do not hold the other party strictly to the letter of the contract, and instead cooperate to agree compromise solutions that keep the NHS market working in difficult times.

## The internal market as a hybrid

For Macneil the relational norms are important in all contractual relations, including within the private sector. Of course, the NHS differs in important ways from the ordinary business world, not least because government requires that purchasers and providers enter contracts with local partners, and that the parties continue to be subject to hierarchical governance (bureaucratic command) as well as what is agreed in the contracts. The typical NHS contract is not a freely-entered agreement, subject to possible non-renewal, and it co-exists with other bureaucratic commitments (such as annual operating frameworks) that limit the parties’ space for manoeuvre.

Building on Oliver Williamson’s analysis of markets and hierarchies [11,12], much recent scholarship has examined the intermediate governance forms that may have developed, including the mix of centralised and decentralised instruments used to control public-sector quasi-markets [14]. A considerable body of scholarly research completed since 1991 has described an NHS ‘managed market’, subject to considerable governmental control and shaped by associated systems of accountability and performance management [15-17]. Like the 1990s internal market, the English and Welsh systems of the early 2000s were hybrids, which combined market-based and command-and-control mechanisms. Contracts, statutory duties, and voluntary action all played a part, and structural or organisational differences between the two countries was also beginning to translate into differences in contracting practices.

In England there were bigger roles for arm’s-length bodies, private providers and consumer choice [18,19]. Regulation depended on a mix of central and decentralised controls operating differentially across the system, so that, for example, while old-style NHS trusts were ‘performance managed’ by the Strategic Health Authorities, the foundation hospitals were overseen by the arm’s-length regulator, Monitor. PCTs remained subject to hierarchical command from both the department and SHA, impeding the full transfer of regulatory functions to Monitor and the Care Quality Commission [20]. On the one hand there was a policy drive to promote competition by developing legally enforceable contracts between NHS purchasers and corporatized foundation trusts, private sector and third sector providers, as well as soft contracts with conventional trusts. But on the other hand providers had to take account of departmental guidance which defined the framework within which these market mechanisms would operate.

In 2007 a programme was launched to support the vision of ‘world class commissioning’ and promote a more strategic, long-term and community-focused approach to commissioning services [21]. Alongside developing skills in areas such as needs assessment and priority setting, ‘world class commissioners effectively stimulate the market to meet demand and secure required clinical, and health and well-being outcomes’ [21][p.21]. Revised principles and rules for co-operation and competition (PRCC) were published which required providers to select the ‘best’ providers on the basis of quality and best-value [22]. As the study progressed, quality improvement was emphasised as one of the key functions of commissioning. A *Commissioning for Quality and Innovation* (CQUIN) initiative [23] was implemented in 2009/10, involving a payment-for-performance scheme under which a small percentage of a provider's income depended on outcomes for patients.

The Welsh Government was less enthusiastic about the market mechanism and did not emulate England’s move towards greater provider pluralism, choice and arm’s length regulation. Instead of relying on oversight by CQC, Wales opted to establish Healthcare Inspectorate Wales, a conventional inspectorate located within a government department. The commissioning process in Wales remained subject to considerable top-down control by the Department of Health and Social Services (DHSS) and its three regional offices, albeit with a concurrent emphasis on localism and the ability of the then 22 LHBs to adapt local plans to local circumstances. Just as in England, although for different reasons, there was a mix of top-down steering and decentralised decision-making, which led to different outcomes in different localities.

By 2007 the Welsh NHS was using softer versions of commissioning based largely of ideas about ‘collegiate contracting’ and partnership working. Welsh purchasers still purchased treatments via NHS contracts, but did so within a framework which also emphasized planning. The LHB’s contracts – its Long Term Agreements (LTAs) - meshed with an Annual Operating Framework (AOF) agreed with the regional office. This in turn was aligned with the Health, Social Care and Well-being Strategies agreed with local government. Thus the horizontal service contract was nested in a set of vertical performance agreements between LHBs or hospital trusts and regional offices, which were enforceable through hierarchical management processes. During the study period Wales introduced a system of regional commissioning in which groups of purchasing LHBs were expected to coordinate their purchasing from local providers.

The differences observed in the four case study localities mostly related to areas of contracting policy that had been influenced by the English NHS’s turn back to markets. English commissioners purchased mainly on PbR tariffs and Welsh LHBs continued to negotiate local prices, and this was reflected in the latter’s greater use of clauses dealing with marginal pricing in contracts. While the use of financial incentives linked to CQUIN was gaining momentum in England, the reverse was happening in Wales, with the demise of the All Wales Sanctions and Incentives Framework. Financial penalties were an important tool supporting targets in England, but were not implemented in Welsh LTAs. The Welsh regional offices operated with a broader conception of performance management than the SHAs, facilitating a three-way negotiation with LHBs and trusts leading to signing off the local annual operating frameworks, compared with a narrower focus on enforcing targets in England.

But these differences existed alongside large areas of common ground. Both systems required purchasers to base their service contracts on national model contracts [24], which mandated the inclusion of certain standard contract terms, and both in practice allowed some scope for a flexible interpretation of these requirements when compliance with the standard clauses caused difficulties. Wales introduced rather more complex arbitration arrangements than England, with the dual tracks of the NHS contracts disputes settlement process and a ‘debtors arbitration’ process, but overall the Welsh and English systems operated in broadly similar ways. The overseeing bodies in both countries actively discouraged contractual disputes, and put considerable pressure on purchasers and providers contemplating use of formal arbitration to negotiate compromise settlements.

## Purchaser/provider relationships in Wales

Where England emphasised competition, Wales introduced organisational arrangements – collegiate contracting and the later regional contracting framework – that recognised the importance of long-term relationships. Under both arrangements the emphasis was on co-operation and long-term understandings between purchasers and providers in a local area. Distant purchasers needing to buy services from a provider in another region were expected to do so by liaising with a lead purchaser in that region, normally using the quality specifications and other requirements already agreed by that local purchaser. Thus rather than finding itself in the position of a buyer completing a one-off transaction with an unfamiliar partner, the distant LHB could benefit from the ongoing relationships built up by the local lead purchaser.

This top-down push towards co-operation did not mean that adversarial behaviour was unknown in the Wales. In fact, the commissioning climate had changed markedly over the lifetime of the LHBs. A study of health authority contracting completed by one of the authors in the 1990s [25] found a cycle of adjustment in which more adversarial contracting relations gave way to more co-operative ones, and this pattern was repeated after the establishment of LHBs. In the period from 2003 to 2006, contracting styles took a reportedly adversarial and combative tone. According to respondents, purchaser/ provider relations became more strained for several reasons: an ‘immaturity’ and lack of capacity in commissioning organisations, an absence of clear policy guidance on the funding responsibilities of secondary and tertiary commissioners (following the establishment of Health Commission Wales), and the pressure on commissioners to achieve financial break-even.

Partly because of a perception that there were too many disputes in the system, the DHSS introduced a model LTA in 2007, followed by new guidance on commissioning which foreshadowed the establishment of three regional commissioning support units (RCSUs). In addition, the financial planning regime was changed so that the Service and Financial Framework (SaFF), which had existed from 2002-03, was replaced by an Annual Operating Framework (AOF). The model LTA, though not mandatory in all its particulars, helped to bring more consistency in contracting practices and ended a situation where documentation had often been unsatisfactory or incomplete. The regional units were intended to encourage co-operation among LHBs, enabling them to negotiate on equal terms with trusts and achieve lower transaction costs. Policy makers aimed to encourage a more strategic approach to service planning associated with the structured tri-annual planning system advocated in the WAG’s *Designed for Life* strategy [26].

The Welsh approach remained softer than the English ‘targets and terror’ regime (a term that refers to the strong management action, including sackings of senior staff, taken to ensure compliance with central NHS targets) [27]. Partly under the influence of the Wanless Report [28], Welsh policy makers had emphasised the need for a rounded assessment of performance that combined use of targets with audit and inspection, including self-assessment. Nevertheless, the pressure on senior managers increased after 2008-09 when each NHS organisation was required to agree an AOF with the regional office containing national and local targets. The Gamma trust finance director told us that he found himself ‘concentrating on waiting times numbers more than you are concentrating on the contract activity levels’. On the commissioner side, LHB finance directors needed to work on developing service specifications linked to AOF targets.

In one of the two Welsh case studies relations between the LHB and main local trust had become very difficult in the year prior to the research, largely because of a protracted dispute about the interpretation of a contract clause on marginal pricing. It was only when one of the main protagonists (the LHB finance director) moved elsewhere that relations improved. Subsequently the LHB rewrote the contract clause concerned with marginal payment rates to rectify an ambiguity exposed by the case.

More generally a number of LHB respondents expressed frustration with a system that combined top-down command with an implied ability to utilise contractual levers. They complained that the effectiveness of such levers was undermined by the unwillingness of the DHSS to support mechanisms such as penalties or arbitration. For example, a number of commissioners recounted their experiences in relation to small block contracts with distant providers. Typically, the value of these contracts had been set by the resource allocation exercise that determined the monies allocated to LHBs when they were established. But some LHBs had reviewed these contracts and determined that they were overpaying distant providers when compared with the prices charged by local providers for the same treatments. They lobbied the DHSS to replace the block contracts with cost per case contracts to reduce costs, but found it unwilling to risk destabilising the financial viability of the trusts by making this change.

The DHSS also sought to limit the use of contractual penalties and formal arbitration in contract disputes, fearing that these would encourage the adversarial relationships that had caused problems in the past. One LHB finance director told us: ‘[There were] potential penalties...I’m struggling to remember what they were, but basically we were told anecdotally don’t implement them, you won’t be supported.’ All the LHBs interviewed had discontinued use of penalty clauses, including penalties for long waiting times which had been common in an earlier period, by the time of the study. Many respondents painted a picture of growing co-operation at this time: as one trust chief executive put it: ‘If you ain’t got enough money to come to the table to buy what you are ready to buy, then there is going to have to be a goodwill basis’.

It might be hypothesized that the Welsh planned economy would lead to a more prescriptive approach towards use of standard contracts compared to England. This was not supported by our data. Several commissioners made minor adjustments to the all-Wales long-term agreement (LTA) and one trust finance director explained that the standard template had simply not been followed in LTAs with the main provider, mainly on the basis of unsuitability in the local context:

*We did our own one which was based very much around concentrating on what we had to.... what we were doing together how we were working together and just recognising that there needed to be sensible dialogue and sensible cut off points in the year that we could use to measure how we were doing against the contract. But we had to allow some flexibility in it on both sides.* (Delta trust3, finance director)

The final days of the internal market in Wales saw an acknowledgement that contracts would now only have a brief life in the transition to a system based on planning, and the running down of the negotiating and monitoring arrangements built up in previous years. This was a period when many meetings were cancelled and tasks that had been carried out in team forums were instead handled in one-to-one meetings between chief executives or finance directors. It was not so much that cooperative behaviour increased *per se*, but that LHB staff knew they would soon be subsumed into unified bodies and became more willing to compromise on contractual requirements. Although guidance required NHS organisations to enter cost-and-volume contracts for most services, purchasers realised that there would be little possibility of holding providers accountable for non-performance and in consequence many of these contracts took a loose ‘block’ form. This led to a simplification of contractual documents, with little attention paid to issues like marginal pricing, and floors and ceilings set so wide as to be nominal.

In Wales, commissioners had moved towards softer forms of contracting over several years. However, rather than developing organically, the relationships between purchasers and providers were further transformed when in 2008 a new Welsh coalition government announced its intention to end the internal market. The final stages of fieldwork saw a kind of forced collaboration between neighbouring LHBs, and between LHBs and trusts. Purchasers no longer talked of contracts as instruments that could be used to lever service improvements and, though the language of partnership and co-operation was much in evidence, this was not so much a reflection of the growth of relational contract norms but the diminished importance of the contracting framework.

## Relationality in England

Conversely the hardening of the English market did not result in a uniform trend towards more adversarial relationships. In both case studies, apparently friendly relations at the personal level co-existed with a significant degree of toughness and tension in the contracting process. Most significantly we found clear examples of cooperative behaviour at junctures in the contracting round when serious financial problems appeared imminent. Even in one case where relationships appeared to worsen in the second year of fieldwork (2009-10), there was a willingness to interpret contractual terms flexibly if a serious problem seemed likely to lead to intervention from the overseeing SHA.

In case study Alpha in Year 1 (2008-09) the meetings we observed were generally conducted in good humour and differences were resolved amicably. Nevertheless, disagreements arose over certain issues that came very close to arbitration, with participants seeking advice from the SHA and in at least one instance from lawyers. During Year 2, there were significant changes in contracting personnel, especially on the trust side. This, together with escalating issues relating to the trust’s data accuracy, led to relationships between the PCT and the trust becoming more formal and adversarial.

Alpha PCT staff complained that the trust was not delivering adequately on a number of contractual requirements, especially those related to data accuracy and transparency. Failure to provide credible or detailed data concerning activity on the part of the trust left the parties unable to reach agreement on activity forecasting and therefore on the overall contract value. The PCT felt that the trust was stubbornly refusing to acknowledge the poor quality of the data returned:

*The problems were to do with the quality of the data from the trust*, *and in my opinion*, *I recognise it’s very much a one-sided opinion*, *we could have avoided quite a lot of the issues if there’d been an acknowledgement up front by the trust that actually the data was… you know… wasn’t good. Because that was the situation we got to in June-July*, *and actually we could have been there a lot earlier. So that certainly didn’t help. Also the financial pressures that the trust were under obviously hindered things. And I think perhaps there’s not a history of openness between the two organisations that might have helped us to speed things along a bit.* (Alpha PCT, finance director)

After a number of successful data challenges by the PCT, the trust’s new finance director warned that cutting the contractual payment would endanger the trust’s financial viability. After further negotiations, and taking account of the trust’s financial problems, the parties agreed to sign a ‘block contract’ containing less stringent data requirements. In the words of Alpha PCT’s finance director : ‘at least we had certainty having the block contract, so there was no risk associated with that contract…it was affordable and it capped our risk, so we decided, you know, perhaps in the circumstances it was a price worth paying, if you like’. By the end of the study the trust was improving its contract information system and efforts were being made on both sides to rebuild relationships.

Relationships in case study Beta ranged from civil and formal to very friendly. Close personal relationships between finance directors proved on occasion very helpful in reaching a mutually acceptable deal for their respective trusts.

One feature of this case study was the difficult financial position in which one PCT found itself. Because Beta PCT2 was in a healthy financial position, while Beta PCT1 was not, Beta trust3 found that only one partner PCT could afford to fund planned service developments. This also meant that Beta PCT1 was hesitant to sign up to jointly-agreed treatment protocols that might have increased its costs, and trust staff became concerned about postcode-based differences in the services provided.

It might be assumed that less problematic finances led to better relationships and respondents indeed reported that relations between Beta PCT2 and Beta trust3 were generally good. But even here there were areas of tension. Trust respondents suggested that the PCT’s management of the contracting processes has been weak, manifested for example in its failure to put in place a proper structure for the contracting cycle. The PCT in turn had been frustrated with the trust’s slowness in submitting data (an issue that resulted in threats to go to arbitration):

*I do think there’s* (*...*) *at a deeper level*, *a lot of mistrust in terms of sharing information...I mean the view from people here is that for a long time*, *the [trust] have been very hesitant about sharing information and data...At a deeper level there is a lot of*, *well*, *we feel they’re sometimes covering up things and not being quite as open as they should be.* (Beta PCT2, assistant director of contracting and performance).

Nevertheless local circumstances bound these two organizations together. Beta trust3 was the single major secondary and tertiary provider in Beta PCT2’s catchment area, so that there was a strong incentive to maintain a cooperative relationship. The option of purchasing services elsewhere made less sense than in a situation where a PCT was close to multiple competing acute trusts. Likewise, the trust’s viability depended on the support of its main purchaser. This mutual dependency meant that the two organisations were locked into a relationship where non-co-operation was not an option.

*When you look at local health economies*, *the situations are all very different*, *so if you have a local health economy that primarily just consists of one PCT and one trust*, *you know*, *and for the trust*, *85% of his income comes from the PCT; and for the PCT*, *85% of his expenditure goes to the trust*, *you’ve got no choice but to work in harmony. Because*, *unless you think that somebody outside is going to come and bail you out*, *those two organisations have got to sort it out.* (Beta trust3, finance director).

This inter-dependency also applied to the financially-strapped Beta PCT1 and its main provider Beta trust2. In a situation where the PCT was unable to pay for an unanticipated surge in activity, the two organisations agreed to relax the PbR rules so that the trust was paid at what were effectively marginal rates rather than according to tariff. Respondents suggested that this was because of the perceived importance of protecting the solvency of their commissioner, which was also in the trust’s long-term interest:

*I mean*, *we’ve always tried to adopt*, *I hope*, *the principle that we want to work with them and not against them. I don’t like confrontation. There is absolutely no advantage for this hospital*, *for them to be in financial difficulty. So*, *we want to work with them. We don’t want to bankrupt them.* (Beta trust2, Finance Director)

In England, the *de facto* interdependency of local partners in a managed market, especially against the background of the reluctance of Monitor and the SHAs to allow formal arbitration, pushed organisations towards co-operation. This was so even in a contracting round where a degree of conflict was manifest. Although this may be viewed as a kind of ‘forced’ relationality, the compromise settlements that kept the market on track would have been hard to achieve without personal relationships across the purchaser/provider split between actors who had known each other for many years. When things went wrong, especially in situations where financial difficulties threatened to de-stabilise a PCT or trust, the smoothing effect of social relations was evident.

## Conclusion

The internal market systems that had developed in England and Wales by the late 2000s differed in their specifics, but both involved a mix of conflictual and co-operative behaviour. Both systems allowed for the use of harder-edged market levers such as contractual penalties and incentives, although English policy makers were strengthening these levers at just the time they were falling into disuse in Wales. Crucially, however, the ultimate market lever of ‘exit’ - ending an exchange relationship and finding a new contracting partner - was not available to NHS organisations. In most cases purchasers and providers were bound together by geography, history and patient expectations (people wished to use their local hospital). Taken together with the reluctance of the overseeing authorities to allow contractual disputes to progress to arbitration, this bilateral interdependency forced local purchasers and providers to find their own solutions, sometimes bending the rules to do so. The compromises achieved generally emerged from negotiations within relational networks that had developed over a long period. While a case study investigation like this one can only suggest, rather than prove, the importance of relationality, the propositions about interdependency and co-operative behaviour put forward here are consistent with the findings of other recent studies, such as those by Exworthy et al. and Porter et al in this supplement.

These findings have implications for the latest NHS reforms now being implemented in England. In our English case studies we found examples where rules were bent to safeguard the financial situation of both a PCT and a trust. Probably many frontline actors have countenanced such practices as a way of maintaining a functional service in difficult times, but some policy makers see such self-protective behaviour as an undesirable attempt to buffer organisations from market levers that would otherwise improve efficiency. In our view the latest reforms in England, which will sweep away the layers of management associated with the PCTs and SHAs, rest on a perception that too many actors in these organisations were willing covertly to resist and undermine the English provider market that Government was seeking to introduce.

Yet there must be real doubts about whether a reform model inspired by one approach within micro-economics – principal-agent theory and related prescriptions for the proper design of incentives – can deliver improvements over the existing system. As Macneil and others have argued, relationality is a crucial feature of contracting in ordinary commercial markets. An English NHS characterised by increased provider pluralism, an influx of new commissioning experts, an exodus of experienced managers and an increasing number of ‘first-time’ (and possible one-off) contracts with new contracting partners, is at odds with theory from socio-legal studies and institutional economics predicting that certain services are best delivered within long-term relationships [12,29]. The dismantling of existing relational networks poses the real risk that a mechanism that allows flexibility in times of trouble will not be there if things go wrong. We suggested earlier that the NHS of the late 2000s was characterised by a kind of ‘forced’ relationality created by the particular dynamics of the managed market of the time, but nevertheless supported by interpersonal contacts built up over many years. In an English NHS populated by Clinical Commissioning Groups, commissioning support agencies, a plurality of providers, the NHS Commissioning Board and the regulators, there will be times when things go wrong, and pressures will grow to again build bridges and turn to co-operative behaviour, but this time in circumstances where informal service networks and organisational memory have been severely damaged.

## Competing interests

The authors declare that they have no competing interests.

## Authors’ contributions

DH was lead author. He developed the conceptual framework for this paper and assisted in Welsh data collection. PA supervised English data collection and helped develop the theoretical framework; SD collected the bulk of the Welsh data and assisted in analysis; CP collected the bulk of the English data and assisted in analysis; PVT worked on the theoretical framework as applied to contracting.

## Declarations

Publication of this supplement was funded by the Nuffield Trust.

This article has been published as part of *BMC Health Services Research* Volume 13 Supplement 1, 2013: The Limits of Market-based Reforms. The full contents of the supplement are available online at http://www.biomedcentral.com/bmchealthservres/supplements/13/S1.
